# Supplementary material for: Genome-Wide Association Study Reveals Genetic Architecture and Candidate Genes for Yield and Related Traits under Terminal Drought, Combined Heat and Drought in Tropical Maize Germplasm
Source: Genes (Basel). 2022 Feb 15;13(2):349. doi: 10.3390/genes13020349 (PMC8871853; doi:10.3390/genes13020349)
Supplement: Supplementary file 1 [file genes-13-00349-s001.zip › Supplementary Fig S1.pdf]

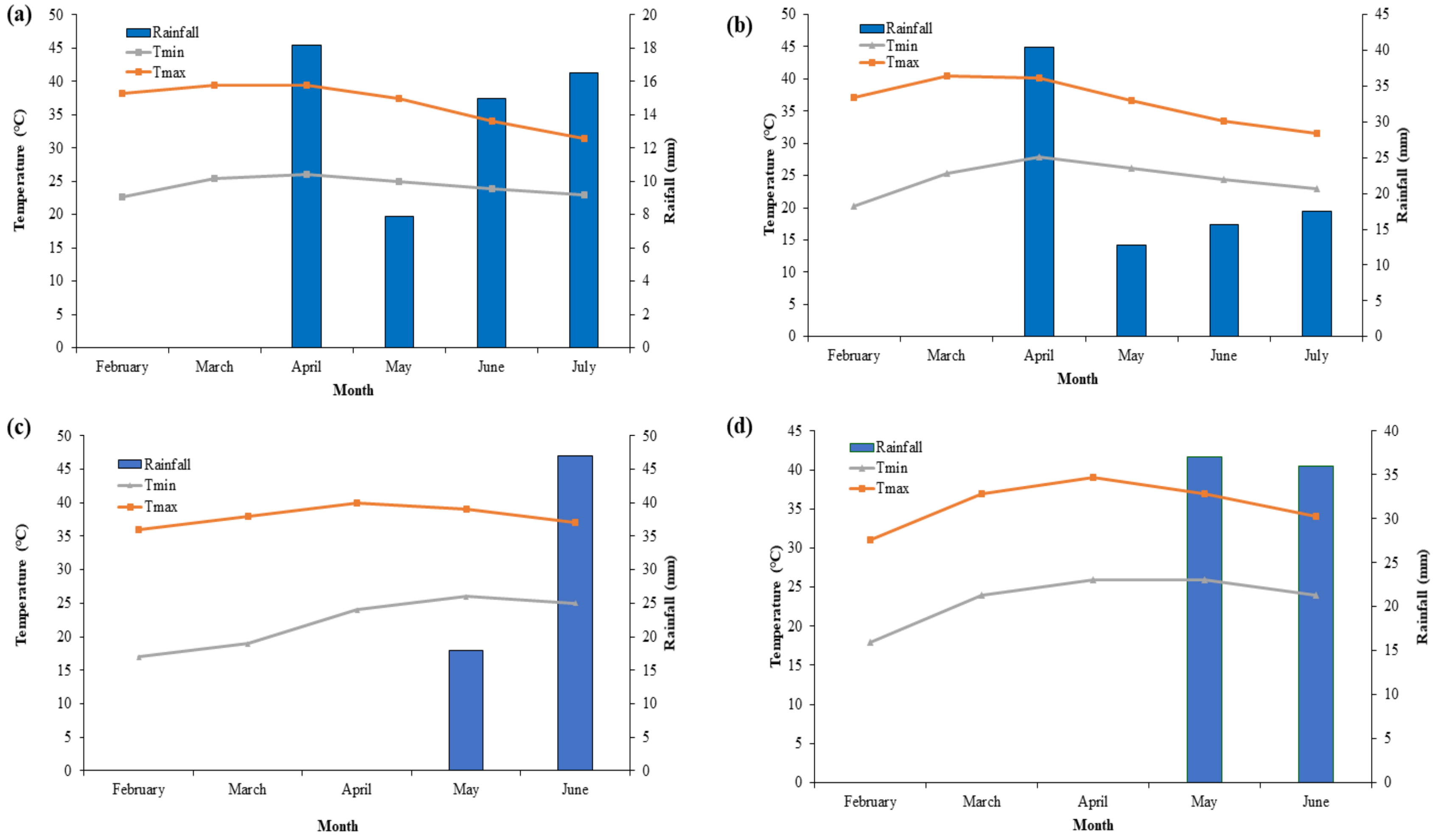


Supplementary Figure S1. Average rainfall and temperature in the two locations during the 2018 and 2019 cropping seasons. (**a**). Manga, Ghana in 2018. (**b**). Manga, Ghana in 2019. (**c**). Kadawa, Nigeria in 2018. (**d**). Kadawa, Nigeria in 2019. Tmin = Minimum Temperature, Tmax. = Maximum Temperature, ºC = Degrees Celsius
