# Supplementary material for: Genome-Wide Association Study Reveals Genetic Architecture and Candidate Genes for Yield and Related Traits under Terminal Drought, Combined Heat and Drought in Tropical Maize Germplasm
Source: Genes (Basel). 2022 Feb 15;13(2):349. doi: 10.3390/genes13020349 (PMC8871853; doi:10.3390/genes13020349)
Supplement: Supplementary file 1 [file genes-13-00349-s001.zip › Supplementary Fig S2.pdf]

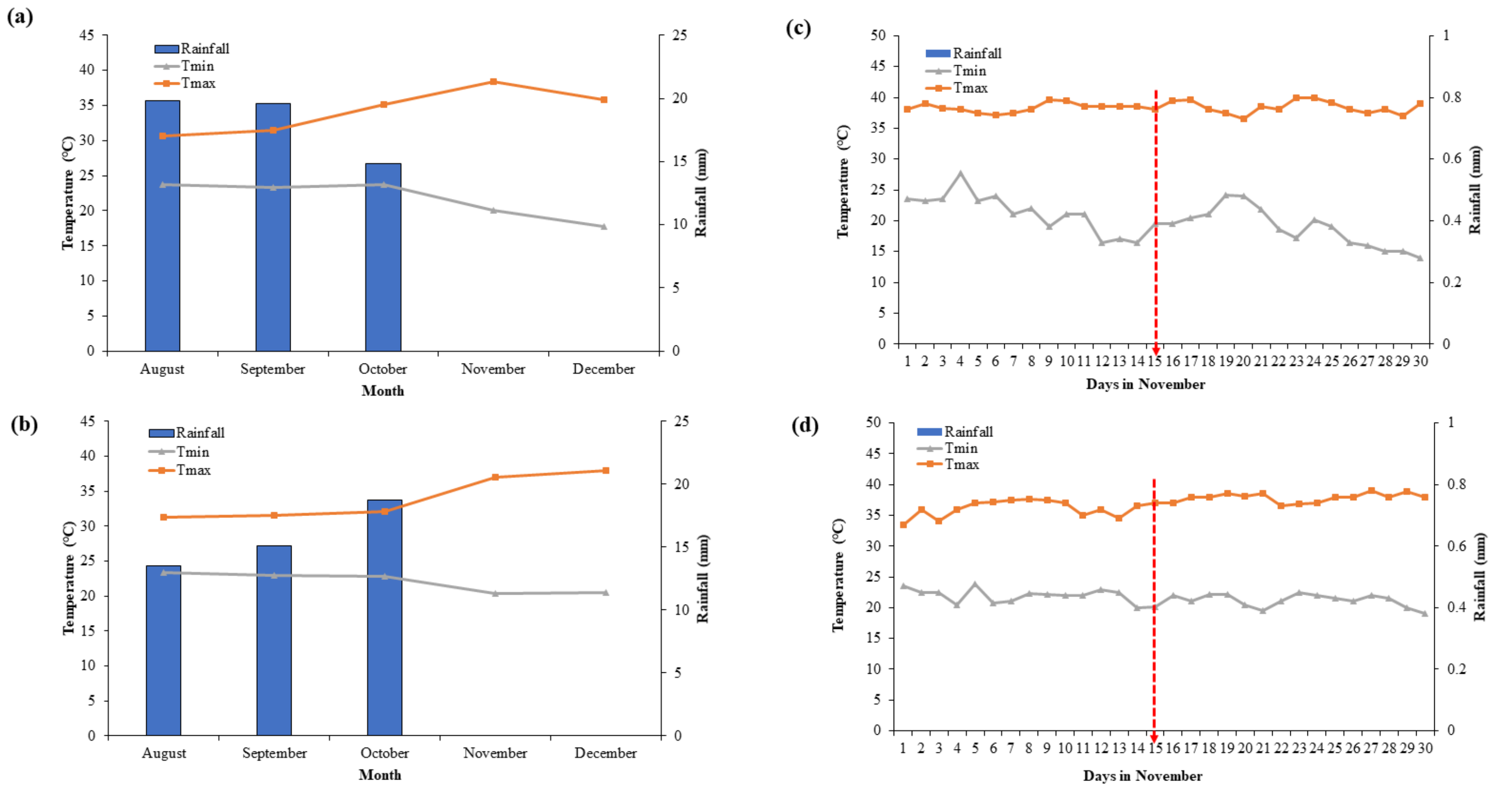

Supplementary Figure S2. Rainfall and temperature readings in Manga during the terminal drought season for the 2018 and 2019 cropping season. (**a**). Manga, Ghana in 2018. (**b**). Manga, Ghana in 2019. (**c**). Daily rainfall and temperature readings in Manga 2018 prior and during tasseling. (**d**). Daily rainfall and temperature readings in Manga 2019 prior and during tasseling. The red dotted line in c and d represent tassel initiation time. Tmin = Minimum Temperature, Tmax. = Maximum Temperature, ºC = Degrees Celsius
