# Supplementary material for: Genome-Wide Association Study Reveals Genetic Architecture and Candidate Genes for Yield and Related Traits under Terminal Drought, Combined Heat and Drought in Tropical Maize Germplasm
Source: Genes (Basel). 2022 Feb 15;13(2):349. doi: 10.3390/genes13020349 (PMC8871853; doi:10.3390/genes13020349)
Supplement: Supplementary file 1 [file genes-13-00349-s001.zip › Supplementary Fig S4.pdf]

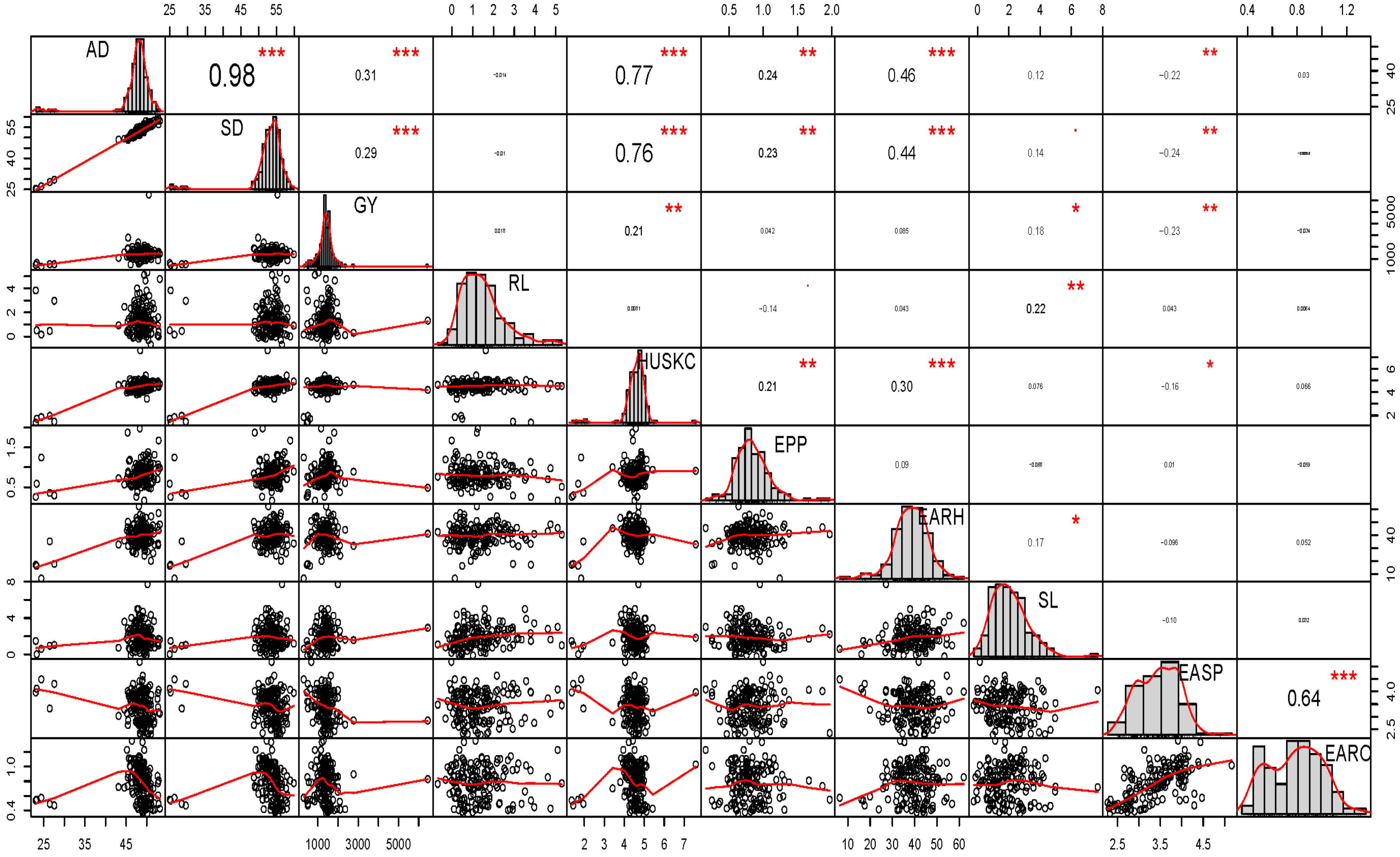

Supplementary Figure S4: Correlation analysis of traits evaluated across the combined heat and drought conditions with average values from Manga (Ghana) and Kadawa (Nigeria). *, ** and *** are significant correlation at $p < 0.05$, $p < 0.01$, $p < 0.001$, respectively. Days to 50% anthesis (AD); days to 50% silking (SD); grain yield (GY); stalk lodging (SL); ear aspect (EASP); Ear rot (EARO); leaf firing (LF); tassel blasting (TB); ear per plant (EPP) and leaf death (LD).
