# Supplementary material for: Genome-Wide Association Study Reveals Genetic Architecture and Candidate Genes for Yield and Related Traits under Terminal Drought, Combined Heat and Drought in Tropical Maize Germplasm
Source: Genes (Basel). 2022 Feb 15;13(2):349. doi: 10.3390/genes13020349 (PMC8871853; doi:10.3390/genes13020349)
Supplement: Supplementary file 1 [file genes-13-00349-s001.zip › Supplementary Fig S5.pdf]

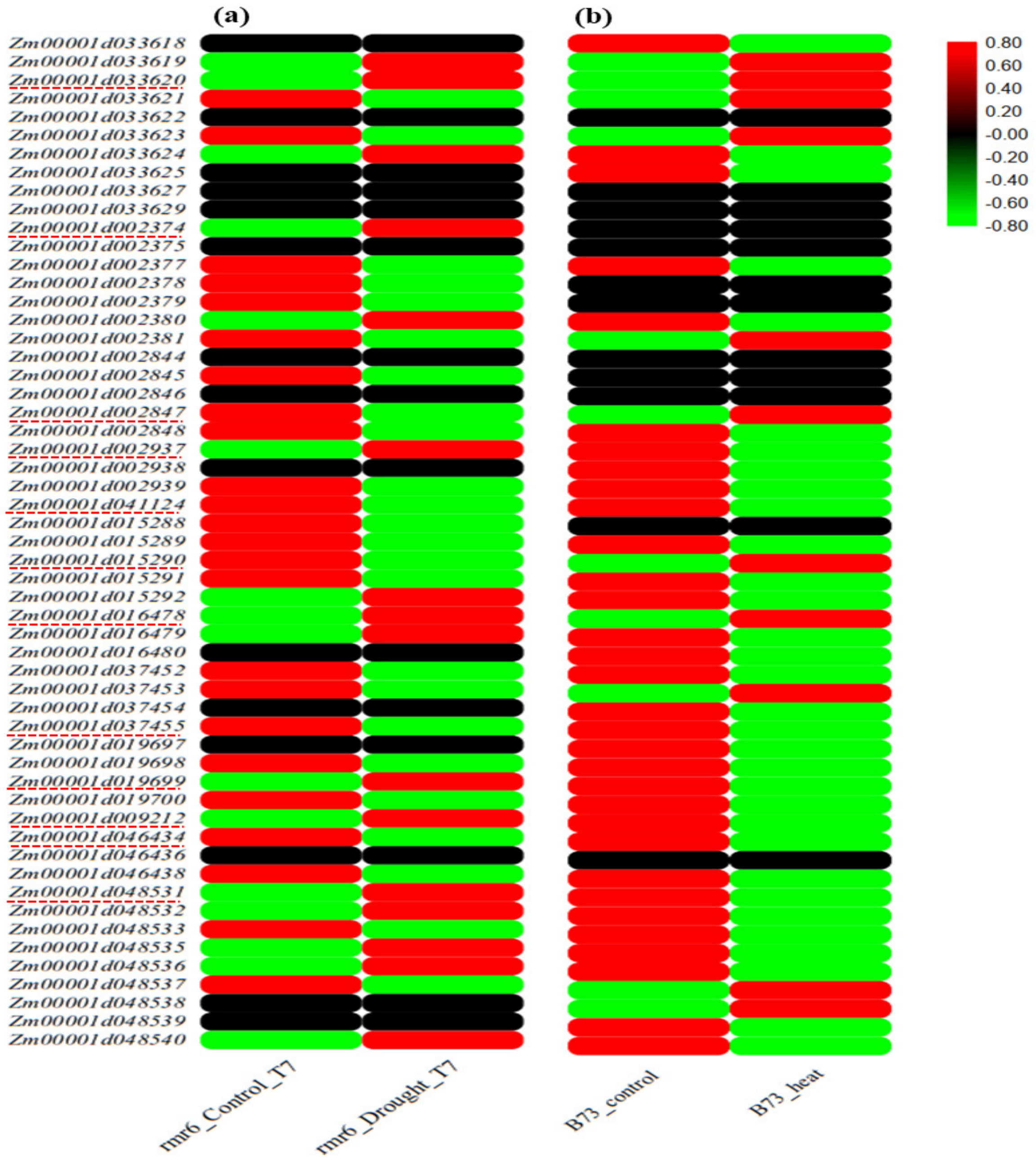

Supplementary Figure S5. Expression of the 55 model genes with the SNP positions ± 120 kb of the markers linked to common traits listed in Table 3. (**a**). Expression of the genes in leaf tissue under drought and control condition with T7 maize cultivar used by Forestan et al. (2016). (**b**). Expression of the genes in leaf tissue under heat and control condition with B73 maize cultivar used by Waters et al. (2017). The genes with dotted red lines are predicted candidate genes based on their annotations retrieved from https://phytozome-next.jgi.doe.gov/ (accessed on 1 January 2022) (version 13). The color legend is shown on right side of the figure (Green and red mean down- and up-regulated, respectively, while the black indicate zero expression)

Forestan, C., R. Aiese Cigliano, S. Farinati, A. Lunardon, W. Sanseverino & S. Varotto, 2016. Stress-induced and epigenetic-mediated maize transcriptome regulation study by means of transcriptome reannotation and differential expression analysis. Scientific reports 6:30446-30446 https://doi.org/10.1038/srep30446.

Waters, A. J., I. Makarevitch, J. Noshay, L. T. Burghardt, C. N. Hirsch, C. D. Hirsch & N. M. Springer, 2017. Natural variation for gene expression responses to abiotic stress in maize. The Plant Journal 89(4):706-717 https://doi.org/10.1111/tpj.13414.
